# General Anesthesia versus Local Anesthesia in StereotaXY (GALAXY) for Parkinson’s disease: study protocol for a randomized controlled trial

**DOI:** 10.1186/s13063-017-2136-8

**Published:** 2017-09-07

**Authors:** R. A. Holewijn, D. Verbaan, R. M. A. de Bie, P. R. Schuurman

**Affiliations:** 1 Department of Neurosurgery, Academic Medical Center, PO Box 22660, 1100 DD Amsterdam, The Netherlands; 2 Department of Neurology, Academic Medical Center, PO Box 22660, 1100 DD Amsterdam, The Netherlands

**Keywords:** Neurosurgery, Parkinson’s disease, Deep brain stimulation, Prospective randomized open-label blinded endpoint trial

## Abstract

**Background:**

The aim of the study is to investigate if deep brain stimulation (DBS) in the subthalamic nucleus (STN) for Parkinson’s disease (PD) under general anesthesia further improves outcome by lessening postoperative cognitive, mood, and behavioral adverse effects; shorten surgical time and hospital admittance; and produce comparable symptomatic and functional improvement to surgery under local anesthesia.

**Methods/design:**

The study will be a single-center, prospective, randomized, open-label, blinded endpoint trial comparing DBS under general anesthesia with DBS under local anesthesia. The primary outcome measure is a composite score of the postoperative cognitive, mood, and behavioral adverse effects and will be measured 6 months after surgery. The secondary outcome measures consist of changes in motor symptoms, adverse effects of stimulation and surgical complications, surgical time, functional health, quality of life, patient satisfaction with the outcome of treatment, patient evaluation of the burden of therapy, and medication. A total of 110 patients with advanced PD who are candidates for DBS will be randomized during a 2.5-year period.

**Discussion:**

The aim of this trial is to further enhance the effectiveness of DBS treatment in PD while reducing the burden of DBS surgery by studying if DBS surgery under general anesthesia results in less cognitive, mood, and behavioral adverse effects compared with surgery under local anesthesia.

**Trial registration:**

Netherlands Trial Register, NTR5809. Registered on 23 April 2016.

**Electronic supplementary material:**

The online version of this article (doi:10.1186/s13063-017-2136-8) contains supplementary material, which is available to authorized users.

## Background

Continuous deep brain stimulation (DBS) of the subthalamic nucleus (STN) is an effective surgical treatment for patients with advanced Parkinson’s disease (PD) who have severe limitations in functioning despite optimal pharmacological treatment [1–5]. Currently, the standard DBS procedure is performed under local anesthesia, which is very burdensome for patients. Due to advances in modern imaging techniques, it is now possible to visualize the DBS target directly [6–9]. Surgery for DBS could therefore be performed under general anesthesia. The literature shows an overall comparable outcome measured with the Unified Parkinson’s Disease Rating Scale (UPDRS [10]) and Movement Disorder Society-sponsored revision of the UPDRS (MDS-UPDRS [11]) motor score in STN DBS under general anesthesia and STN DBS under local anesthesia [12–22]. There is little evidence about the rate of adverse events in STN DBS under general anesthesia [17, 18, 21]. Furthermore, the effect of general anesthesia on cognitive, mood, and behavioral adverse effects is poorly investigated [21, 23]. STN DBS under local anesthesia is very burdensome for all patients and holds back some who are actually good candidates for the procedure. We hypothesize that STN DBS under general anesthesia would be much less traumatic for patients and would lead to less cognitive, mood, and behavioral adverse effects. To our knowledge, no randomized controlled trial has been done to compare the outcome of STN DBS in patients who undergo the surgery under general anesthesia versus local anesthesia. We aim to investigate whether STN DBS under general anesthesia will further improve outcome by lessening postoperative cognitive, mood, and behavioral adverse effects 6 months after surgery; shorten surgical time and hospital admittance; and produce comparable symptomatic and functional improvement to that of surgery under local anesthesia.

## Methods/design

### Trial design

In this study, using a prospective, randomized, open-label, blinded endpoint (PROBE) study design, we will assess the effectiveness of STN DBS surgery under general anesthesia compared with the current standard practice of surgery under local anesthesia. The study schedule is shown in Additional file 1: Figure S1.

### Setting

The protocol of this single-center study was approved by the Medical Ethics Committee of the Academic Medical Center (AMC), Amsterdam, The Netherlands. It is registered with the Netherlands Trial Register (NTR5809). Inclusion and randomization of the study started on May 23, 2016.

### Participants

The inclusion criteria are (a) idiopathic PD with bradykinesia and at least two of the following signs: resting tremor, rigidity, and/or asymmetry; (b) despite optimal pharmacological treatment, at least one of the following symptoms: severe response fluctuations, dyskinesias, painful dystonia, or bradykinesia; (c) older than 18 years of age; and (d) a life expectancy of at least 2 years. Exclusion criteria are (a) legally incompetent adults; (b) previous PD-related neurosurgery (e.g., DBS, pallidotomy, thalamotomy); (c) contraindications for DBS surgery, such as a physical disorder making surgery hazardous; (d) Hoehn and Yahr stage 5 at the best moment during the day; (e) current psychosis; (f) current depression; and (g) no written informed consent.

### Study procedures

Patients are referred to the AMC by their treating neurologist for the indication of DBS. According to regular DBS care, the patients are admitted for several days to evaluate the following: disease severity; response to PD medication; and cognition, mood, and behavior. After this screening, the patient is discussed in a multidisciplinary meeting with the neurologist, neurosurgeon, neuropsychologist, psychiatrist, and specialized nurse in order to decide if the patient is eligible for surgery.

When the patient is a good candidate for DBS surgery, the attending neurologist/neurosurgeon will check inclusion and exclusion criteria for the study. If the patient is eligible, the physician coordinating the study will inform the patient, hand over written information about the trial, explain the trial to the patient, and subsequently ask the patient to consider participation. The consent procedure is further described below under the “Ethics approval and consent to participate” heading. After signing the informed consent form, the patient will be randomized.

### Randomization

The randomization procedure will be website-based using random blocks and stratified by the response to a suprathreshold levodopa dose administered in off-drug phase. This response is measured as the ratio of the MDS-UPDRS part III score in on-/off-drug phases. The response to levodopa is a good indicator of the effect of DBS. A cutoff of 40% improvement in MDS-UPDRS in on-drug phase was used to separate the two stratification groups. The cutoff is based on the median improvement of patients treated with DBS in the STN in a previous nationwide study on the effectiveness of DBS in patients with PD [24]. The web-based method of randomization will provide concealment allocation and prevent the risk of selection bias. Participants will be randomized using TENALEA, which is an online, centralized randomization service. Allocation concealment will be ensured because the service will not release the randomization code until the patient has been recruited into the trial, which takes place after all baseline measurements have been completed, as shown in Additional file 1: Figure S1.

### Intervention

DBS treatment is performed by a team that consists of various specialties (e.g., neurologist, neurosurgeon, specialized nurses). Treatment is in accordance with the usual care regarding this procedure. A neurosurgeon places two electrodes in the brain. These are connected to an implantable pulse generator. All patients will receive anesthetics during placement of the pulse generator. Currently, patients are hospitalized for 4 days, on average. On the day of surgery, patients do not receive PD drugs until the end of the procedure.

#### Local anesthesia

The stereotactic technique will be employed for implantation with the Leksell stereotactic frame (Elekta, Stockholm, Sweden) and guided by magnetic resonance imaging (MRI). For this part of the surgery, patients will have the stereotactic frame attached to the head under local anesthesia, after which an MRI scan is obtained and the implantation of electrodes is carried out. The decision for electrode placement is based on MRI scan, microelectrode recordings (three channels), and intraoperative macroelectrode stimulation effects. Two electrodes will be implanted, one in each STN. Subsequently, patients will have a second surgery under general anesthesia to implant the pulse generator subcutaneously in the subclavian area. The electrodes are connected to the pulse generator.

#### General anesthesia

The stereotactic technique will be employed for implantation with the Leksell stereotactic frame (Elekta, Stockholm, Sweden) and guided by MRI. Patients receive general anesthesia before the stereotactic frame is fitted to the head. The decision for electrode placement is based on MRI and microelectrode recordings (two to three channels). In contrast to the local anesthesia group, no macroelectrode stimulation will be performed. The electrodes will be implanted into the STN. In the same procedure, patients will undergo implantation of the pulse generator subcutaneously in the subclavian area. The electrodes are connected to the pulse generator.

During follow-up, the use of oral comedication is allowed in both groups just as in regular daily practice and current DBS treatment. The treating neurologist supervises any changes in medication. The main motivation for this approach is to stay close to regular daily practice routines for reasons of generalizability.

### Outcome measures

#### Primary outcome measure

The primary outcome measure is the number of patients with significant postoperative cognitive, mood, and behavioral adverse effects as indicated by a stringent composite score of ≥ 1 within 6 months after STN DBS. Significant cognitive, mood, and behavioral adverse effects are defined in four areas: (a) deterioration of cognition in three domains or more would lead to assigning a point for cognition on the composite endpoint; a Reliable Change Index less than or equal to −1.645 on at least one subtest per domain would lead to the conclusion of deterioration in this domain [25]; (b) loss of professional activity, work, or job; (c) postoperative delirium assessed with the Confusion Assessment Method [26] and the Delirium Observation Scale [27]; or (d) psychosis, depression, or anxiety for a period of 3 months or longer as defined by the Mini International Neuropsychiatric Interview psychiatric assessment [28] (Additional file 2: Table S1).

#### Secondary outcome measures

Secondary endpoints are the efficacy of treatment on the following:

- Motor symptoms as measured by MDS-UPDRS part III and the Clinical Dyskinesia Rating Scale [29]
- Daily functioning measured with MDS-UPDRS part II
- Nonmotor experiences of daily living as measured by MDS-UPDRS part I
- Functional health status measured with the Academic Medical Center Linear Disability Score [30]
- Quality of life measured with the 39-item Parkinson’s Disease Questionnaire (PDQ-39) [31]
- Treatment satisfaction
- Burden of therapy
- Surgical time
- Duration of hospital admittance
- Surgery- and stimulation-related side effects
- PD medication changes
- Other motor complications measured with UPDRS part IV
- Other psychiatric adverse effects as measured with the Hamilton Depression Rating Scale [32], Hamilton Anxiety Rating Scale [33], Columbia Suicide Severity Rating Scale [34], Starkstein Apathy Scale [35], and Young Mania Rating Scale [36]

*See* Additional file 3: Table S2 for further details.

### Sample size calculation

We estimate that by performing DBS surgery under general anesthesia, a relative reduction of 50% in cognitive, mood, and behavioral adverse effects will be achieved. To detect a relative reduction of 50% (52% local anesthesia versus 26% general anesthesia) using a chi-square test with an α value of 0.05 (two-sided) and β value of 0.2, we estimated that 110 patients (55 in each treatment group) would be needed. We expect adequate participant enrollment to reach our target sample size because we operate on 80 patients with PD per year. Furthermore, in our previous clinical trials, we have always been able to reach an inclusion ratio of 70% when studying patients with PD.

### Statistical analysis

Statistical analyses will be based on the intention-to-treat principle. Baseline assessments and outcome parameters will be summarized using simple descriptive statistics. Change scores are calculated as the score at follow-up minus the score at baseline. To test the hypothesis that STN DBS under general anesthesia produces fewer patients with significant postoperative cognitive, mood, and behavioral adverse effects, the primary outcome score will be compared using a chi-square test. Adjustments for factors that differ at randomization, including the stratification variable, will be made using regression or multilevel models. Effect sizes will be expressed as ORs. We will perform additional per-protocol and as-treated analyses.

The mean change in MDS-UPDRS Part III scores from baseline to follow-up at 6 months will be analyzed using a two-group *t* test. With regard to the comparisons of the other secondary outcomes, we will use the appropriate parametric and nonparametric statistics. Additionally, we will focus on the MDS-UPDRS part III scores at 6 months using multivariable linear regression, including the stratification variable and other factors that differ at randomization, in the model. In all analyses, statistical uncertainty will be expressed with 95% CIs. *P* values < 0.05 will be considered statistically significant. We will perform additional per-protocol and as-treated analyses. A fully specified statistical analysis plan will be written and approved before unlocking the data blinding, provided in the form of a *Trials* Update article.

### Data safety analysis

An interim analysis will be performed by a data and safety monitoring board (DSMB) when half of the patients (*n* = 55) are enrolled. In this analysis, unblinded data are assessed, and the DSMB can advise on adjusting the study conduct, design, or sample size or on whether to terminate the study. The justifications for a recommendation to terminate the study due to clear harm will be based on data showing a notable increase of (serious) adverse events (including cognitive, mood, and behavioral adverse effects) in the general anesthesia group.

## Discussion

Currently, patients need to be awake during surgery to corroborate optimal placement of the stimulation electrodes in the brain. This procedure is very burdensome for patients. Their antiparkinson medication is withheld overnight, which renders their parkinsonism very severe during the operation. A metal frame is fixed to their head, after which electrodes are implanted in a lengthy procedure during which the patients are continuously tested for the effect of DBS. Due to advances in MRI techniques, the target for DBS can now be clearly visualized, and therefore it may be possible to perform the entire procedure under general anesthesia, without the need for intraoperative clinical testing.

Changing the operative strategy for DBS from local anesthesia to general anesthesia could make the therapy more effective by (a) reducing the burden of the procedure, (b) reducing the immediate postoperative side effects, and (c) increasing the efficiency of the operation. Furthermore, it would make the therapy accessible to those patients who are currently judged to be unable to withstand the procedure because of their generally weakened medical or borderline abnormal cognitive condition. Also, patients for whom DBS surgery under local anesthesia is currently not possible due to severe spinal deformities or due to severe dystonic posturing can be good candidates for surgery under general anesthesia. This will expand the target patient group that can benefit from DBS treatment.

Some of the choices in the study design warrant discussion. First, is the study design. Current literature comparing STN DBS under general anesthesia with STN DBS under local anesthesia consists of case series, case-control studies, or cohort studies [12–22]. The strength of our study is the randomization into two groups, which will provide a higher level of evidence. In this surgical trial, it is impossible to blind the neurosurgeon and/or patient to treatment assignment. In order to apply blinding where possible, the study will be conducted using a PROBE design. The assessors of the primary and secondary endpoints will be blinded. Patients will be instructed not to reveal the received treatment to the trial nurse performing the postoperative assessments or to the psychiatrist and the neuropsychologist performing (respectively) the psychiatric and neuropsychological evaluation, in order to secure a blinded endpoint.

Second are the primary and secondary outcome measures. In contrast to researchers in most other DBS trials, we chose the primary endpoint as a stringent composite score of cognitive, mood, and behavioral effects. This score was shown to be of importance in a previous national DBS study comparing internal globus pallidus DBS with STN DBS in patients with PD [24]. The composite score is a dichotomous measure and assesses cognitive, mood, and behavioral adverse effects after DBS surgery. In contrast to the NSTAPS study investigators [24], we have chosen to exclude the parameter “loss of an important relationship” because this does not necessarily imply worsening of disability or perceived quality of life. Instead, we included the parameter delirium because this is a frequent known postoperative complication in patients with PD receiving DBS surgery. The composite score will specifically address clinical measures to determine cognitive, mood, and behavioral effects, and therefore it has an advantage regarding use of a more general quality-of-life scale as a primary outcome measure. In order to be able to compare our results with those of other trials, the widely used PDQ-39 will be used in the secondary outcome measurements. Our secondary outcome measurements will further show the motor function, complications, adverse events, surgical time, duration of hospital admittance, medication, and patient satisfaction and burden of therapy.

Third is the sample size motivation. Up to one-third of our patients currently treated under local anesthesia experience moderate to severe postoperative confusion due to the heavy burden of undergoing a long surgery, and their necessary dopaminergic medication is withheld overnight in order to judge the effect of stimulation on their symptoms during the procedure. Although this postoperative confusion creates an additional burden that sometimes needs to be managed medically and leads to longer hospital admittance, this in itself may not necessarily have a negative influence on the long-term outcome of surgery. However, in the current practice of awake surgery, considerable exploration in the target area is performed to determine the location for implantation, which we believe is associated with the current incidence of postoperative cognitive, affective, and behavioral adverse effects. This is supported by some reports describing that the postoperative decline in cognition, when it occurs, is not dependent on active stimulation being applied, and therefore it is believed to be caused by the mechanical impact of surgery itself rather than by the application of chronic stimulation afterward [37–39]. Electrode implantation under general anesthesia will be focused more on the imaging to determine the location for implantation, with the support of microelectrode recordings in the predetermined implantation trajectory. This strategy will lead to considerably less intraoperative exploration in the STN of the majority of patients and therefore will likely be associated with less postoperative sequelae. We therefore estimate that, by performing DBS surgery under general anesthesia, a relative reduction of 50% in cognitive, mood, and behavioral adverse effects may be achieved.

In conclusion, we have developed a protocol for a prospective, randomized, open-label study of STN DBS under general anesthesia. Our study will provide safety and efficacy results on STN DBS under general anesthesia compared with the current standard, STN DBS with local anesthesia.

### Trial status

The date of first enrollment was May 23, 2016. The estimated study duration will be 2.5 years. Participants are currently being recruited and enrolled.

## Additional files

Additional file 1: Figure S1. Study schedule. (DOCX 31 kb)

Additional file 2: Table S1. Composite score. Legend: ^a^: standard of care. (DOC 64 kb)

Additional file 3: Table S2. Baseline characteristics and secondary outcome measures. Legend: ^a^ Standard of care. ^b^ MDS-UPDRS part III in four conditions: condition 1 - medication off, stimulation on; condition 2 – medication off, stimulation off; condition 3 – medication on, stimulation off; condition 4 – medication on, stimulation on. (DOC 81 kb)

Additional file 4: Figure S2. SPIRIT schedule of enrollment, interventions, and assessments. (DOC 122 kb)

Additional file 5: SPIRIT 2013 checklist: recommended items to address in a clinical trial protocol and related documents. (DOC 86 kb)

## Abbreviations

AMC: Academic Medical Center
DBS: Deep brain stimulation
DSMB: Data and safety monitoring board
MDS-UPDRS: Modified Unified Parkinson’s Disease Rating Scale
METC: Medisch Ethische Toetsingscommissie (Medical Ethical Committee)
MRI: Magnetic resonance imaging
PD: Parkinson’s disease
PI: Principal investigator
PROBE: Prospective, randomized, open-label, blinded endpoint
STN: Subthalamic nucleus
UPDRS: Unified Parkinson’s Disease Rating Scale

## References

[1] Deuschl G, Schade-Brittinger C, Krack P, Volkmann J, Schafer H, Botzel K, Daniels C, Deutschlander A, Dillmann U, Eisner W (2006). A randomized trial of deep-brain stimulation for Parkinson’s disease. N Engl J Med..

[2] Weaver FM, Follett K, Stern M, Hur K, Harris C, Marks WJ, Rothlind J, Sagher O, Reda D, Moy CS (2009). Bilateral deep brain stimulation vs best medical therapy for patients with advanced Parkinson disease: a randomized controlled trial. JAMA..

[3] Kleiner-Fisman G, Herzog J, Fisman DN, Tamma F, Lyons KE, Pahwa R, Lang AE, Deuschl G (2006). Subthalamic nucleus deep brain stimulation: summary and meta-analysis of outcomes. Mov Disord..

[4] Williams A, Gill S, Varma T, Jenkinson C, Quinn N, Mitchell R, Scott R, Ives N, Rick C, Daniels J (2010). Deep brain stimulation plus best medical therapy versus best medical therapy alone for advanced Parkinson’s disease (PD SURG trial): a randomised, open-label trial. Lancet Neurol..

[5] Krack P, Batir A, Van Blercom N, Chabardes S, Fraix V, Ardouin C, Koudsie A, Limousin PD, Benazzouz A, LeBas JF (2003). Five-year follow-up of bilateral stimulation of the subthalamic nucleus in advanced Parkinson’s disease. N Engl J Med..

[6] Brunenberg EJ, Platel B, Hofman PA, Ter Haar Romeny BM, Visser-Vandewalle V (2011). Magnetic resonance imaging techniques for visualization of the subthalamic nucleus. J Neurosurg..

[7] Patil PG, Conrad EC, Aldridge JW, Chenevert TL, Chou KL (2012). The anatomical and electrophysiological subthalamic nucleus visualized by 3-T magnetic resonance imaging. Neurosurgery..

[8] Slavin KV, Thulborn KR, Wess C, Nersesyan H (2006). Direct visualization of the human subthalamic nucleus with 3 T MR imaging. AJNR Am J Neuroradiol..

[9] Cheng CH, Huang HM, Lin HL, Chiou SM (2014). 1.5 T versus 3 T MRI for targeting subthalamic nucleus for deep brain stimulation. Br J Neurosurg.

[10] Movement Disorder Society Task Force on Rating Scales for Parkinson’s Disease (2003). The Unified Parkinson’s Disease Rating Scale (UPDRS): status and recommendations. Mov Disord.

[11] Goetz CG, Tilley BC, Shaftman SR, Stebbins GT, Fahn S, Martinez-Martin P, Poewe W, Sampaio C, Stern MB, Dodel R (2008). Movement Disorder Society-sponsored revision of the Unified Parkinson’s Disease Rating Scale (MDS-UPDRS): scale presentation and clinimetric testing results. Mov Disord..

[12] Hertel F, Zuchner M, Weimar I, Gemmar P, Noll B, Bettag M, Decker C (2006). Implantation of electrodes for deep brain stimulation of the subthalamic nucleus in advanced Parkinson’s disease with the aid of intraoperative microrecording under general anesthesia. Neurosurgery..

[13] Yamada K, Goto S, Kuratsu J, Matsuzaki K, Tamura T, Nagahiro S, Murase N, Shimazu H, Kaji R (2007). Stereotactic surgery for subthalamic nucleus stimulation under general anesthesia: a retrospective evaluation of Japanese patients with Parkinson’s disease. Parkinsonism Relat Disord..

[14] Nakajima T, Zrinzo L, Foltynie T, Olmos IA, Taylor C, Hariz MI, Limousin P (2011). MRI-guided subthalamic nucleus deep brain stimulation without microelectrode recording: can we dispense with surgery under local anaesthesia?. Stereotact Funct Neurosurg..

[15] Patel NK, Heywood P, O’Sullivan K, Love S, Gill SS (2002). MRI-directed subthalamic nucleus surgery for Parkinson’s disease. Stereotact Funct Neurosurg..

[16] Foltynie T, Zrinzo L, Martinez-Torres I, Tripoliti E, Petersen E, Holl E, Aviles-Olmos I, Jahanshahi M, Hariz M, Limousin P (2011). MRI-guided STN DBS in Parkinson’s disease without microelectrode recording: efficacy and safety. J Neurol Neurosurg Psychiatry..

[17] Harries AM, Kausar J, Roberts SA, Mocroft AP, Hodson JA, Pall HS, Mitchell RD (2012). Deep brain stimulation of the subthalamic nucleus for advanced Parkinson disease using general anesthesia: long-term results. J Neurosurg..

[18] Moll CK, Payer S, Gulberti A, Sharrott A, Zittel S, Boelmans K, Koppen J, Gerloff C, Westphal M, Engel AK (2013). STN stimulation in general anaesthesia: evidence beyond ‘evidence-based medicine’. Acta Neurochir Suppl..

[19] Lefaucheur JP, Gurruchaga JM, Pollin B, von Raison F, Mohsen N, Shin M, Menard-Lefaucheur I, Oshino S, Kishima H, Fenelon G (2008). Outcome of bilateral subthalamic nucleus stimulation in the treatment of Parkinson’s disease: correlation with intra-operative multi-unit recordings but not with the type of anaesthesia. Eur Neurol..

[20] Sutcliffe AJ, Mitchell RD, Gan YC, Mocroft AP, Nightingale P (2011). General anaesthesia for deep brain stimulator electrode insertion in Parkinson’s disease. Acta Neurochir (Wien).

[21] Chen SY, Tsai ST, Lin SH, Chen TY, Hung HY, Lee CW, Wang WH, Chen SP, Lin SZ (2011). Subthalamic deep brain stimulation in Parkinson’s disease under different anesthetic modalities: a comparative cohort study. Stereotact Funct Neurosurg..

[22] Lettieri C, Rinaldo S, Devigili G, Pauletto G, Verriello L, Budai R, Fadiga L, Oliynyk A, Mondani M, D’Auria S (2012). Deep brain stimulation: subthalamic nucleus electrophysiological activity in awake and anesthetized patients. Clin Neurophysiol..

[23] Fluchere F, Witjas T, Eusebio A, Bruder N, Giorgi R, Leveque M, Peragut JC, Azulay JP, Regis J (2014). Controlled general anaesthesia for subthalamic nucleus stimulation in Parkinson’s disease. J Neurol Neurosurg Psychiatry..

[24] Odekerken VJ, van Laar T, Staal MJ, Mosch A, Hoffmann CF, Nijssen PC, Beute GN, van Vugt JP, Lenders MW, Contarino MF (2013). Subthalamic nucleus versus globus pallidus bilateral deep brain stimulation for advanced Parkinson’s disease (NSTAPS study): a randomised controlled trial. Lancet Neurol..

[25] Jacobson NS, Truax P (1991). Clinical significance: a statistical approach to defining meaningful change in psychotherapy research. J Consult Clin Psychol..

[26] Inouye SK, van Dyck CH, Alessi CA, Balkin S, Siegal AP, Horwitz RI (1990). Clarifying confusion: the confusion assessment method. A new method for detection of delirium. Ann Intern Med.

[27] Schuurmans MJ, Shortridge-Baggett LM, Duursma SA (2003). The Delirium Observation Screening Scale: a screening instrument for delirium. Res Theory Nurs Pract..

[28] Sheehan DV, Lecrubier Y, Sheehan KH, Amorim P, Janavs J, Weiller E, Hergueta T, Baker R, Dunbar GC (1998). The Mini-International Neuropsychiatric Interview (M.I.N.I.): the development and validation of a structured diagnostic psychiatric interview for DSM-IV and ICD-10. J Clin Psychiatry.

[29] Hagell P, Widner H (1999). Clinical rating of dyskinesias in Parkinson’s disease: use and reliability of a new rating scale. Mov Disord..

[30] Weisscher N, Post B, de Haan RJ, Glas CA, Speelman JD, Vermeulen M (2007). The AMC Linear Disability Score in patients with newly diagnosed Parkinson disease. Neurology..

[31] Marinus J, Visser M, Jenkinson C, Stiggelbout AM (2008). Evaluation of the Dutch version of the Parkinson’s Disease Questionnaire 39. Parkinsonism Relat Disord..

[32] Hamilton M (1960). A rating scale for depression. J Neurol Neurosurg Psychiatry..

[33] Hamilton M (1959). The assessment of anxiety states by rating. Br J Med Psychol..

[34] Posner K, Brown GK, Stanley B, Brent DA, Yershova KV, Oquendo MA, Currier GW, Melvin GA, Greenhill L, Shen S, Mann JJ (2011). The Columbia-Suicide Severity Rating Scale: initial validity and internal consistency findings from three multisite studies with adolescents and adults. Am J Psychiatry..

[35] Starkstein SE, Mayberg HS, Preziosi TJ, Andrezejewski P, Leiguarda R, Robinson RG (1992). Reliability, validity, and clinical correlates of apathy in Parkinson’s disease. J Neuropsychiatry Clin Neurosci..

[36] Young RC, Biggs JT, Ziegler VE, Meyer DA (1978). A rating scale for mania: reliability, validity and sensitivity. Br J Psychiatry..

[37] Yilmaz R, Akbostanci MC, Mercan FN, Sorgun MH, Savas A (2015). No effect of different stimulation conditions on verbal fluency and visuospatial orientation in patients with subthalamic nucleus deep brain stimulation. Stereotact Funct Neurosurg..

[38] Pillon B, Ardouin C, Damier P, Krack P, Houeto JL, Klinger H, Bonnet AM, Pollak P, Benabid AL, Agid Y (2000). Neuropsychological changes between “off” and “on” STN or GPi stimulation in Parkinson’s disease. Neurology..

[39] Okun MS, Gallo BV, Mandybur G, Jagid J, Foote KD, Revilla FJ, Alterman R, Jankovic J, Simpson R, Junn F (2012). Subthalamic deep brain stimulation with a constant-current device in Parkinson’s disease: an open-label randomised controlled trial. Lancet Neurol..

