# Supplementary material for: General Anesthesia versus Local Anesthesia in StereotaXY (GALAXY) for Parkinson’s disease: study protocol for a randomized controlled trial
Source: Trials. 2017 Sep 7;18:417. doi: 10.1186/s13063-017-2136-8 (PMC5590197; doi:10.1186/s13063-017-2136-8)
Supplement: Supplementary file 1 — Study schedule. (DOCX 31 kb) [file 13063_2017_2136_MOESM1_ESM.docx]

Neurologist refers patient to AMC

Baseline screening standardized ON and OFF phase: eligible for DBS surgery?

Patient not eligible for DBS surgery

55 patients receive STN DBS under general anesthesia

55 patients receive STN DBS under local anesthesia + pulse generator placement under general anesthesia

Follow-up: day 1 or 2 after surgery, 2 weeks and 6 months

Patient refuses to participate or is refused based on in-/ exclusion criteria

Follow-up: day 1 or 2 after surgery, 2 weeks and 6 months

Standard STN DBS under local anesthesia

Neurologist/ neurosurgeon AMC checks in- and exclusion criteria, the study coordinating physician introduces the study and provides written information.

Neurosurgeon AMC asks eligible patients informed consent

110 eligible patients willing to be randomized
