# Supplementary material for: General Anesthesia versus Local Anesthesia in StereotaXY (GALAXY) for Parkinson’s disease: study protocol for a randomized controlled trial
Source: Trials. 2017 Sep 7;18:417. doi: 10.1186/s13063-017-2136-8 (PMC5590197; doi:10.1186/s13063-017-2136-8)
Supplement: Supplementary file 2 — Composite score. Legend: a: standard of care. (DOC 64 kb) [file 13063_2017_2136_MOESM2_ESM.doc]

Table 1 - Composite Score

| **Areas** | **Scale** | **Visit 1**  **Baseline screening** | **Visit 2**  **Day 1 or 2 after surgery** | **Visit 3**  **2 weeks after surgery** | **Visit 4**  **6 months after surgery** |
| --- | --- | --- | --- | --- | --- |
| **Cognition** |  |  |  |  |  |
| *Language* | Boston naming Test [1]  Verbal Fluency (subtests: letter) [2] | X^a^ |  |  | X^a^ |
| *Intelligence* | Wechsler Adult Intelligent Scale (WAIS) IV similarity subtest [3]  Dutch reading test Adults [4] | X^a^ |  |  | X^a^ |
| *Memory* | Auditory Verbal Learning Test, Dutch version (15 word test (subtests: total, recall)) [5]  Rivermead Behavioral memory test: stories [6] | X^a^ |  |  | X^a^ |
| *Attention and executive functions* | Trailmaking test (subtest: A, B) [7]  Stroop color-word test [8] | X^a^ |  |  | X^a^ |
| *Complex visual perception* | Judgement of line orientation [9] | X^a^ |  |  | X^a^ |
| *Constructive skills* | Clock [10] | X^a^ |  |  | X^a^ |
| **Professional activity, work or job** |  |  |  |  |  |
|  | Interview | X |  |  | X |
| **Psychosis, depression, anxiety** |  |  |  |  |  |
|  | Psychiatric evaluation (incl. Mini-international neuropsychiatric interview (MINI)) [11] | X |  |  | X |
| **Delirium** |  |  |  |  |  |
|  | Confusion Assessment Method (CAM) [12] |  | X | X |  |
|  | Delirium Observation Scale (DOS) [13] |  | X |  |  |

^a^: standard of care

**References**

1. Kaplan E, Goodglass H, Weintraub S, Brand S: *Boston Naming Test.* Lea & Febiger; 1983.

2. Rosen WG: **Verbal fluency in aging and dementia.** *Journal of Clinical Neuropsychology* 1980, **2:**135-146.

3. Wechsler D: **WAIS-IV administration and scoring manual.** San Antonio, Texas: Pearson; 2008.

4. Mulder J, Bouma, J. M. & Schmand, B.: **Nederlandse leestest voor volwassenen.** In *Handboek neuropsychologische diagnostiek* (Bouma JM, Mulder, J., Lindeboom, J. & Schmand, B., editors ed. pp. 127-138. Amsterdam: Pearson Assessment and Information B.V.; 2012:127-138.

5. Rey A: *L'examen clinique en psychologie.* Paris: Presses universitaires de France; 1964.

6. Wilson BC, J. Baddely, A.: **The Rivermead behavioural memory test.** Reading, UK: Thames Valley Test Company; 1985.

7. Reitan R: **Trail making test manual for administration and scoring.** Tucson, Arizona, USA: Reitan Neuropsychological Laboratory; 1992.

8. Stroop JR: **Studies of interference in serial verbal reactions.** *Journal of Experimental Psychology* 1935, **18:**643.

9. Benton AL, Varney NR, Hamsher KD: **Visuospatial judgment. A clinical test.** *Arch Neurol* 1978, **35:**364-367.

10. Freedman ML, L.; Kaplan, E.; Winocur, G.; Shulman, K.I.; Delis, D.: **Clock Drawing: A Neuropsychological Analysis.** New York: Oxford University Press; 1994.

11. Sheehan DV, Lecrubier Y, Sheehan KH, Amorim P, Janavs J, Weiller E, Hergueta T, Baker R, Dunbar GC: **The Mini-International Neuropsychiatric Interview (M.I.N.I.): the development and validation of a structured diagnostic psychiatric interview for DSM-IV and ICD-10.** *J Clin Psychiatry* 1998, **59 Suppl 20:**22-33;quiz 34-57.

12. Inouye SK, van Dyck CH, Alessi CA, Balkin S, Siegal AP, Horwitz RI: **Clarifying confusion: the confusion assessment method. A new method for detection of delirium.** *Ann Intern Med* 1990, **113:**941-948.

13. Schuurmans MJ, Shortridge-Baggett LM, Duursma SA: **The Delirium Observation Screening Scale: a screening instrument for delirium.** *Res Theory Nurs Pract* 2003, **17:**31-50.
