# Supplementary material for: General Anesthesia versus Local Anesthesia in StereotaXY (GALAXY) for Parkinson’s disease: study protocol for a randomized controlled trial
Source: Trials. 2017 Sep 7;18:417. doi: 10.1186/s13063-017-2136-8 (PMC5590197; doi:10.1186/s13063-017-2136-8)
Supplement: Supplementary file 3 — Baseline characteristics and secondary outcome measures. Legend: a Standard of care. b MDS-UPDRS part III in four conditions: condition 1 - medication off, stimulation on; condition 2 – medication off, stimulation off; condition 3 – medication on, stimulation off; condition 4 – medication on, stimulation on. (DOC 81 kb) [file 13063_2017_2136_MOESM3_ESM.doc]

Table 2 – Baseline characteristics and secondary outcome measures

| **Scale** | **Visit 1**  **Baseline screening** | **Visit 2**  **Day 1 or 2 after surgery** | **Visit 3**  **2 weeks after surgery** | **Visit 4**  **6 months after surgery** |
| --- | --- | --- | --- | --- |
| Demographic characteristics | X^a^ |  |  |  |
| Medication | X^a^ | X^a^ | X^a^ | X^a^ |
| Videotape OFF and ON phase | X^a^ |  |  |  |
| Clinical Dyskinesia Rating Scale (CDRS) [1] | X |  |  | X |
| Hoehn and Yahr stage [2] | X^a^ |  |  | X |
| MDS-UPDRS [3] | X^a^ |  |  | X^b^ |
| AMC linear disability scale (ALDS) [4] | X |  |  | X |
| PDQ-39 [5] | X |  |  | X |
| Hamilton Depression Scale [6] | X |  |  | X |
| Hamilton Anxiety Scale [7] | X |  |  | X |
| Columbia Suicide Severity Rating Scale [8] | X |  |  | X |
| Starkstein Apathy Scale [9] | X |  |  | X |
| Young Mania Rating Scale [10] | X |  |  | X |
| Mattis Dementia Rating Score [11] | X^a^ |  |  | X |
| Dutch readingtest for adults [12] | X^a^ |  |  |  |
| Parkinson’s Disease – Cognitive Rating Scale (PD-CRS) [13] | X^a^ |  |  |  |
| Surgery time |  | X |  |  |
| Hospital admittance duration |  |  | X |  |
| Treatment burden |  | X | X | X |
| Treatment satisfaction |  |  |  | X |
| Side effects/adverse events/complications |  | X | X | X |

^a^: Standard of care.

^b^: MDS-UPDRS part III in four conditions: Condition 1 - Medication OFF, Stimulation ON; Condition 2 – Medication OFF, Stimulation OFF; Condition 3 – Medication ON, Stimulation OFF; Condition 4 – Medication ON, Stimulation ON.

**References**

1. Hagell P, Widner H: **Clinical rating of dyskinesias in Parkinson's disease: use and reliability of a new rating scale.** *Mov Disord* 1999, **14:**448-455.

2. Goetz CG, Tilley BC, Shaftman SR, Stebbins GT, Fahn S, Martinez-Martin P, Poewe W, Sampaio C, Stern MB, Dodel R, et al: **Movement Disorder Society-sponsored revision of the Unified Parkinson's Disease Rating Scale (MDS-UPDRS): scale presentation and clinimetric testing results.** *Mov Disord* 2008, **23:**2129-2170.

3. Movement Disorder Society Task Force on Rating Scales for Parkinson's D: **The Unified Parkinson's Disease Rating Scale (UPDRS): status and recommendations.** *Mov Disord* 2003, **18:**738-750.

4. Weisscher N, Post B, de Haan RJ, Glas CA, Speelman JD, Vermeulen M: **The AMC Linear Disability Score in patients with newly diagnosed Parkinson disease.** *Neurology* 2007, **69:**2155-2161.

5. Marinus J, Visser M, Jenkinson C, Stiggelbout AM: **Evaluation of the Dutch version of the Parkinson's Disease Questionnaire 39.** *Parkinsonism Relat Disord* 2008, **14:**24-27.

6. Hamilton M: **A rating scale for depression.** *J Neurol Neurosurg Psychiatry* 1960, **23:**56-62.

7. Hamilton M: **The assessment of anxiety states by rating.** *Br J Med Psychol* 1959, **32:**50-55.

8. Posner K, Brown GK, Stanley B, Brent DA, Yershova KV, Oquendo MA, Currier GW, Melvin GA, Greenhill L, Shen S, Mann JJ: **The Columbia-Suicide Severity Rating Scale: initial validity and internal consistency findings from three multisite studies with adolescents and adults.** *Am J Psychiatry* 2011, **168:**1266-1277.

9. Starkstein SE, Mayberg HS, Preziosi TJ, Andrezejewski P, Leiguarda R, Robinson RG: **Reliability, validity, and clinical correlates of apathy in Parkinson's disease.** *J Neuropsychiatry Clin Neurosci* 1992, **4:**134-139.

10. Young RC, Biggs JT, Ziegler VE, Meyer DA: **A rating scale for mania: reliability, validity and sensitivity.** *Br J Psychiatry* 1978, **133:**429-435.

11. Mattis S: **Dementia Rating Scale Professional Manual.** Odessa, Florida: Psychological Assessment Resources; 1998.

12. Schmand B LJ, van Harskamp F: **NLV: Nederlandse Leestest voor Volwassenen, Handleiding.** Lisse: Swets & Zeitlinger; 1992.

13. Pagonabarraga J, Kulisevsky J, Llebaria G, Garcia-Sanchez C, Pascual-Sedano B, Gironell A: **Parkinson's disease-cognitive rating scale: a new cognitive scale specific for Parkinson's disease.** *Mov Disord* 2008, **23:**998-1005.
