# Supplementary material for: General Anesthesia versus Local Anesthesia in StereotaXY (GALAXY) for Parkinson’s disease: study protocol for a randomized controlled trial
Source: Trials. 2017 Sep 7;18:417. doi: 10.1186/s13063-017-2136-8 (PMC5590197; doi:10.1186/s13063-017-2136-8)
Supplement: Supplementary file 5 — SPIRIT 2013 checklist: recommended items to address in a clinical trial protocol and related documents. (DOC 86 kb) [file 13063_2017_2136_MOESM5_ESM.doc]

Figure 2 – SPIRIT schedule of enrolment, interventions, and assessments.

|  | **STUDY PERIOD** | | | | | |
| --- | --- | --- | --- | --- | --- | --- |
|  | **Enrolment** | **Allocation** | **Post-allocation** | | | **Close-out** |
| **TIMEPOINT** | ***-t_1_*** | **0** | ***t_1_*** | ***t_2_*** | ***t_3_*** | ***t_x_*** |
|  | ***Pre-study***  ***Screening/***  ***Consent*** |  | **Surgery** | **Day 1 or 2 after surgery** | **2 weeks after surgery** | **6 months after surgery** |
| **ENROLMENT:** |  |  |  |  |  |  |
| **Eligibility screen** | X |  |  |  |  |  |
| **Informed consent** | X |  |  |  |  |  |
| **Allocation** |  | X |  |  |  |  |
| **INTERVENTIONS:** |  |  |  |  |  |  |
| ***DBS under local anesthesia*** |  |  | X |  |  |  |
| ***DBS under general anesthesia*** |  |  | X |  |  |  |
| **ASSESSMENTS:** |  |  |  |  |  |  |
| ***Baseline variables*** |  |  |  |  |  |  |
| Demographic characteristics | X |  |  |  |  |  |
| Medication | X |  |  | X | X | X |
| Videotape OFF and ON phase | X |  |  |  |  |  |
| Clinical Dyskinesia Rating Scale (CDRS) | X |  |  |  |  | X |
| Hoehn and Yahr stage | X |  |  |  |  | X |
| MDS-UPDRS | X |  |  |  |  | X |
| AMC linear disability scale (ALDS) | X |  |  |  |  | X |
| PDQ-39 | X |  |  |  |  | X |
| Hamilton Depression Scale | X |  |  |  |  | X |
| Hamilton Anxiety Scale | X |  |  |  |  | X |
| Columbia Suicide Severity Rating Scale | X |  |  |  |  | X |
| Starkstein Apathy Scale | X |  |  |  |  | X |
| Young Mania Rating Scale | X |  |  |  |  | X |
| Mattis Dementia Rating Score | X |  |  |  |  | X |
| Dutch readingtest for adults | X |  |  |  |  |  |
| Parkinson’s Disease – Cognitive Rating Scale (PD-CRS) | X |  |  |  |  |  |
| Surgery time | X |  |  | X |  |  |
| Hospital admittance duration | X |  |  |  | X |  |
| Treatment burden | X |  |  | X | X | X |
| Treatment satisfaction | X |  |  |  |  | X |
| Side effects/adverse events/complications | X |  |  | X | X | X |
| ***Composite score*** |  |  |  |  |  |  |
| *Cognition* |  |  |  |  |  |  |
| Language  Boston naming Test  Verbal Fluency (subtests: letter) |  |  |  | X |  | X |
| Intelligence  Wechsler Adult Intelligent Scale (WAIS) IV similarity subtest  Dutch reading test Adults |  |  |  | X |  | X |
| Memory  Auditory Verbal Learning Test, Dutch version (15 word test (subtests: total, recall))  Rivermead Behavioral memory test: stories |  |  |  | X |  | X |
| Attention and executive functions  Trailmaking test (subtest: A, B)  Stroop color-word test |  |  |  | X |  | X |
| Complex visual perception  Judgement of line orientation |  |  |  | X |  | X |
| Constructive skills  Clock |  |  |  | X |  | X |
| *Professional activity, work or job* |  |  |  |  |  |  |
| Interview |  |  |  | X |  | X |
| *Psychosis, depression, anxiety* |  |  |  |  |  |  |
| Psychiatric evaluation (incl. Mini-international neuropsychiatric interview (MINI)) |  |  |  | X |  | X |
| *Delirium* |  |  |  |  |  |  |
| Confusion Assessment Method (CAM) |  |  |  | X |  | X |
| Delirium Observation Scale (DOS) |  |  |  | X |  | X |
